# Effectiveness of either short-duration ischemic pre-conditioning, single-set high-resistance exercise, or their combination in potentiating bench press exercise performance

**DOI:** 10.3389/fphys.2022.1083299

**Published:** 2022-12-15

**Authors:** Andreas Salagas, Athanasios Tsoukos, Gerasimos Terzis, Vassilis Paschalis, Christos Katsikas, Michal Krzysztofik, Michal Wilk, Adam Zajac, Gregory C. Bogdanis

**Affiliations:** ^1^ School of Physical Education and Sports Science, National and Kapodistrian University of Athens, Athens, Greece; ^2^ Institute of Sport Sciences, Jerzy Kukuczka Academy of Physical Education in Katowice, Katowice, Poland

**Keywords:** velocity-based training, warm-up, performance enhancement, post-activation potentiation, blood flow restricted exercise

## Abstract

This study compared the effects of short-duration ischemic preconditioning, a single-set high-resistance exercise and their combination on subsequent bench press performance. Twelve men (age: 25.8 ± 6.0 years, bench press 1-RM: 1.21 ± 0.17 kg kg^−1^ body mass) performed four 12 s sets as fast as possible, with 2 min of recovery between sets, against 60% 1-RM, after: a) 5 min ischemic preconditioning (IPC; at 100% of full arterial occlusion pressure), b) one set of three bench press repetitions at 90% 1-RM (PAPE), c) their combination (PAPE + IPC) or d) control (CTRL). Mean barbell velocity in ischemic preconditioning was higher than CTRL (by 6.6–9.0%, *p* < 0.05) from set 1 to set 3, and higher than PAPE in set 1 (by 4.4%, *p* < 0.05). Mean barbell velocity in PAPE was higher than CTRL from set 2 to set 4 (by 6.7–8.9%, *p* < 0.05), while mean barbell velocity in PAPE + IPC was higher than CTRL only in set 1 (+5.8 ± 10.0%). Peak barbell velocity in ischemic preconditioning and PAPE was higher than CTRL (by 7.8% and 8.5%, respectively; *p* < 0.05). Total number of repetitions was similarly increased in all experimental conditions compared with CTRL (by 7.0–7.9%, *p* < 0.05). Rating of perceived exertion was lower in ischemic preconditioning compared with CTRL (*p* < 0.001) and PAPE (*p* = 0.045), respectively. These results highlight the effectiveness of short-duration ischemic preconditioning in increasing bench press performance, and suggest that it may be readily used by strength and conditioning coaches during resistance training due to its brevity and lower perceived exertion.

## Introduction

Two of the most common pre-conditioning methods used by athletes and coaches are the post-activation performance enhancement (PAPE) and ischemic pre-conditioning (IPC) (Kilduff et al., 2013; Blazevich and Babault, 2019; Krzysztofik et al., 2020, 2021; Wilk et al., 2021). PAPE has been defined as an acute enhancement of muscle performance following pre-conditioning using a series of maximal or submaximal muscle actions (Tillin and Bishop, 2009; Tsoukos et al., 2016). These muscle actions usually involve resistance or plyometric exercises, and have been shown to acutely increase muscular strength and power (Conrado de Freitas et al., 2021; Tsolakis et al., 2011), movement velocity (Tsoukos et al., 2021a), rate of force development (Arabatzi et al., 2014) and total work of training (Alves et al., 2019). The optimal recovery time between the pre-conditioning exercises and subsequent muscular performance varies from a few seconds (French et al., 2003) to 20 min (Gilbert and Lees, 2005) and depends on the balance between PAPE and fatigue (Rassier and Macintosh, 2000). Previous research has shown that the PAPE effect is greater when using heavy compared with light loads (Rahimi, 2007; Bogdanis et al., 2014; Tsoukos et al., 2019; Tsoukos et al., 2021a; Krzysztofik et al., 2021). However, fatigue is also enhanced when resistance is high (Tsoukos et al., 2021b), and thus a longer recovery time (4–20 min) may be necessary between the pre-conditioning exercise with heavy loads and subsequent performance (Gilbert and Lees, 2005; Tsoukos et al., 2019; Tsoukos et al., 2021a; Krzysztofik et al., 2021). Moreover, improving explosive performance, PAPE has been shown to increase the total number of repetitions, total work, and total time under tension (TUT) during resistance exercise protocols performed to exhaustion (Alves et al., 2019; Krzysztofik et al., 2020). However, limited information exists regarding the effects of PAPE protocols on barbell velocity and total volume during repeated sets performed with maximum intended velocity for a set duration (10–30 s), mimicking high-intensity functional training (HIFT) with free weights which is a popular training modality in gym settings (Feito et al., 2018; Kapsis et al., 2022).

Ischemic preconditioning (IPC) is another pre-conditioning technique which consists of one extended (e.g., several repeated periods or “cycles” (usually 3-4 x 5 min) of ischemia, followed by equal duration reperfusion periods (Sharma et al., 2015). A series of studies found that IPC may increase performance in different exercise modalities where the oxidative or the glycolytic energy systems dominate (Incognito et al., 2016; Salvador et al., 2016). IPC results in enhanced VO_2_ kinetics (Walsh et al., 2002), muscle oxygenation (Kido et al., 2015), power output (Kraus et al., 2015) applied force (Paradis-Deschênes et al., 2016), as well as higher training load (Carvalho and Barroso, 2019). These benefits seem to be observed as a result of greater metabolic efficiency (Murry et al., 1990; Andreas et al., 2011), increased blood flow (Cunniffe et al., 2017) and higher neural activation (de Oliveira Cruz et al., 2017). Few studies have examined the effect of IPC during resistance exercise on barbell velocity, total number of repetitions performed and total volume during resistance exercise, and found improvements (Carvalho and Barroso, 2019; Guilherme Da Silva Telles et al., 2020; da Silva Novaes et al., 2021; Wilk et al., 2021). However, in all these studies the authors used either 4 cycles of 5 min of occlusion at cuff pressure of 220 Hg, alternated by equal periods of reperfusion prior to exercise (Carvalho and Barroso, 2019; Guilherme Da Silva Telles et al., 2020; da Silva Novaes et al., 2021), or occlusion applied during the recovery between sets of resistance exercise with very short reperfusion periods (Wilk et al., 2021). From a practical viewpoint, these approaches require a long period of pre-conditioning before the execution of the exercise sets, or may cause discomfort and performance drop if IPC is used during exercise (Paixão et al., 2014; Cocking et al., 2018). To our knowledge, no study has examined IPC of very brief duration on subsequent performance during resistance training, and it would be of great practical interest if only one cycle of IPC is adequate to cause performance enhancement. Therefore, we examined the effects of: a) a single 5-min period of IPC at full occlusion pressure [100% of full arterial occlusion pressure (AOP)], followed by 5 min of reperfusion, b) a high-resistance PAPE protocol (1 set of 3 repetitions at 90% of 1-RM) and c) the combination of IPC and PAPE protocols, on barbell velocity, training load (total number of repetitions) and rating of perceived exertion during four sets of the bench press exercise against a load of 60% of 1-RM.

## Methods

### Experimental design

**FIGURE 1:**
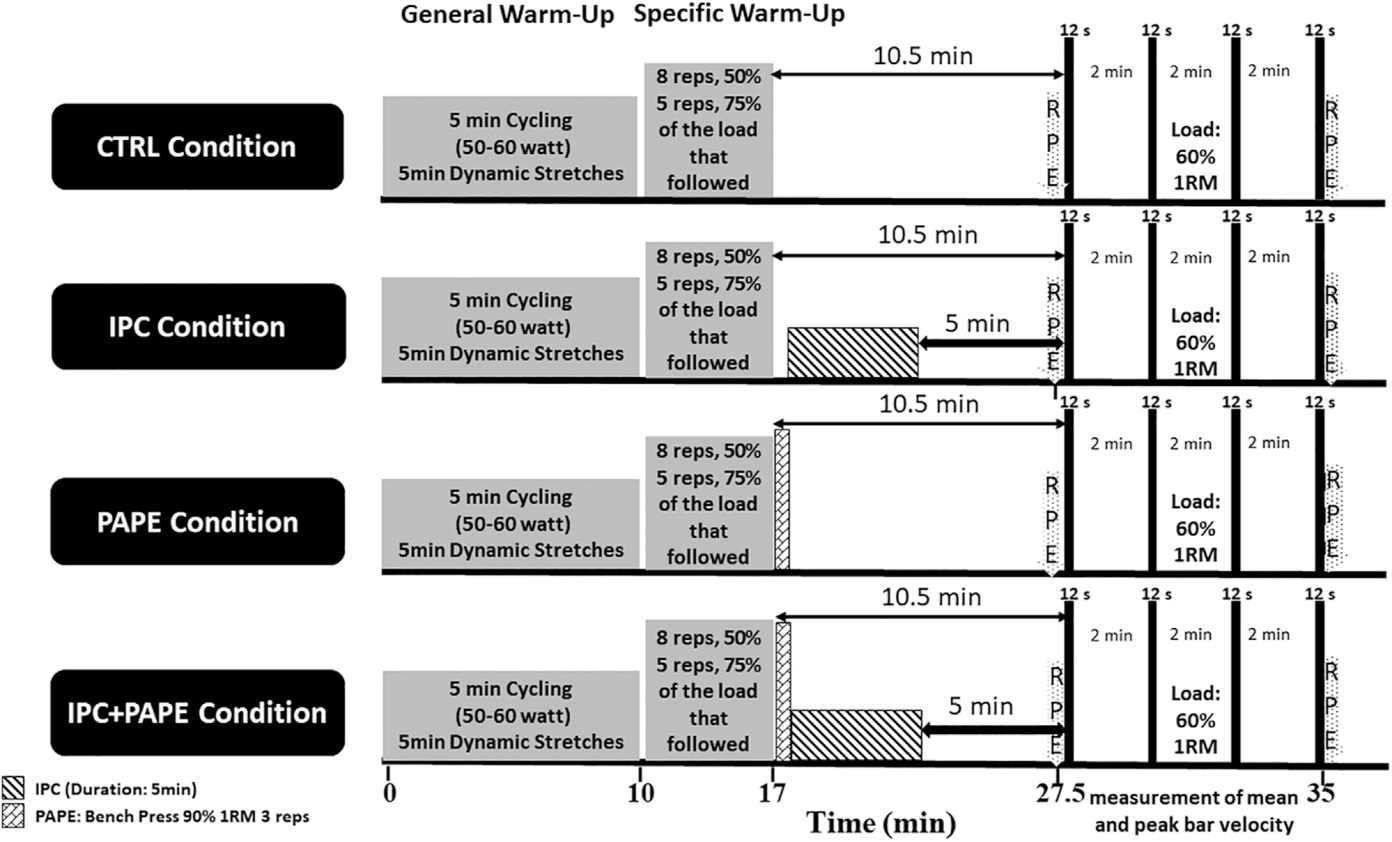
Schematic representation of the experimental design.

A randomized and counterbalanced repeated measures latin square design was used. The participants completed two preliminary sessions, followed by three experimental and one control session, 1 week apart. The three experimental sessions involved short-duration IPC, PAPE, and a combined IPC + PAPE intervention, while during the control condition the participants rested for 10.5 min (Figure 1). After each intervention, subjects performed 4 sets of 12 s duration each, of bench presses executed as fast as possible against a load of 60% of 1-RM on a Smith machine. Each set was followed by 2 min of passive recovery. The 60% of 1-RM load was chosen because it has been shown to combine the characteristics of the high average surface electromyographic (sEMG) activity of heavier loads, and the high total integrated sEMG observed at lighter loads, when sets are executed as fast as possible until exhaustion (Tsoukos et al., 2021b). During the first preliminary visit, anthropometric data were obtained and the maximum dynamic bench press strength (1-RM) was measured. In the second preliminary visit, the individual full arterial occlusion pressure (AOP) was determined, and the participants were familiarized with executing the bench press exercise as fast as possible from the first repetition. The dependent variables were: the average mean barbell velocities of all the repetitions in each set, the average peak barbell velocities of all the repetitions in each set, the total number of repetitions, and the rating of perceived exertion (RPE).

### Subjects

Twelve healthy men participated in the study after completing an informed consent form (age: 25.8 ± 6.0 years, weight: 79.7 ± 8.9 kg, height: 1.82 ± 0.04 m, bench press 1-RM: 95.8 ± 13.3 or 1.21 ± 0.17 kg kg^−1^ body mass). The following inclusion criteria were used to select participants: a) they were healthy and physically active for at least 6 months before the study, b) their bench press 1 RM exceeded their body weight. Exclusion criteria were: a) any musculoskeletal injuries of the upper body for at least 6 months prior to the study, b) any blood or intraocular pressure problems.

The participants were instructed to maintain their habitual dietary routine over the course of the study and to abstain from the use of any supplements or stimulants during the experiment. Before providing their written consent, they were informed about the benefits and the risks of the study, and also about their right to withdraw from the study at any time without providing any explanation. The protocol of the study was approved by the Bioethics Committee of the School of Physical Education and Sport Science of Athens, Greece (1279/14-4-2021), and the experimental procedures were in accordance with the Declaration of Helsinki, 1983.

### Procedures

#### Familiarization session and the 1 RM strength test

Before the main experiment, the participants performed two preliminary sessions. In the first of the two preliminary sessions, anthropometric data were collected, and the 1-RM bench-press strength was measured on a Smith machine. The subjects warmed-up on a cycle ergometer for 5 min at (50–60 W) followed by 5 min upper body dynamic stretching for chest and arms (Tsoukos et al., 2019; Tsoukos et al., 2021a). After the completion of the general warm-up, the participants were instructed to follow the procedure outlined by the National Strength and Conditioning Association (Gregory Haff and Travis Triplett, 2016). The feet were placed flat on the floor with a knee angle of approximately 90 and the head, shoulders and hips were supported by the bench. Assistance was provided throughout the test by two experienced spotters, who were qualified strength coaches. The participants were instructed to grasp the bar with a narrow width at 100% of bi-acromial distance (Barnett et al., 1995). The distance between hands was measured and kept the same for all sessions. During the procedure verbal encouragement was given to all participants. The ICC for the 1-RM measurement in our laboratory is 0.92 (Tsoukos et al., 2021a). Twenty minutes after the completion of the 1-RM test, subjects were familiarized with the occlusion cuffs, which included a manometer (Fit Cuffs Arms, Odder, Denmark, cuff width: 7.5 cm). The cuffs were worn near the axillary’s fossa of both arms, while subjects lay on the bench. Cuffs were inflated to 140 mmHg and this pressure was maintained for 3 min.

In the second preliminary session, the participants wore the occlusion cuffs in close proximity to the axillary’s fossa of both arms, lay on the bench for 10 min, and the individual cuff pressure at 100% of full arterial occlusion (AOP: 146.7 ± 15.0 mmHg) was determined using a pulse oximeter (Contec Holter ABPM50, Contec Medical Systems, Qinhuangdao, Hebei Province, China). This measurement was conducted twice on each arm (total of four times) with a 10 min interval. Ten minutes after the determination of the individual AOP, the participants were familiarized with the ischemic pre-conditioning and the PAPE protocol. Immediately after the completion of the standardized general and specific warm-up (see Figure 1), subjects performed one set of bench press, comprising three repetitions at 90% of 1-RM on a Smith machine. Thirty seconds after the end of warm-up, the cuffs were inflated at 100% AOP for 5 min, followed by a 5-min period of reperfusion. Afterwards, subjects performed two sets of 12 s duration each, at 60% of 1 RM with 2 min rest intervals between them with the intention to move as fast as possible from the first to the last repetition. The intention of movement was required to be maximal for both the eccentric and the concentric phase of the movement for each repetition (Wilk et al., 2020a; Wilk et al., 2020b).

### Measurements

Movement velocity was recorded with a linear position transducer (Tendo Power analyzer System v. 314, TENDO Sports Machines, Trencin, Slovak Republic). The string of the linear position transducer was positioned vertically to the barbell of the Smith machine. Τhe position of the transducer was set up by hanging a small weight from the bar to the floor before the start of any condition. This procedure was done to secure that the vertical velocity of the barbell was measured correctly. The validity and reliability of this system has been presented elsewhere (Garnacho-Castaño et al., 2014). The average of mean barbell velocities (AMV) was determined as the mean value of all mean velocities of the repetitions in each set. The average of peak barbell velocities (APV) (m∙s^−1^) was determined as the mean value of all peak velocities of all repetitions in every set. The ICCs for these measurements in our laboratory are as follows: MV [0.983 (95% CI: 0.962–0.995)] and PV [0.971 (95% CI: 0.932–0.992)] (Tsoukos et al., 2019; Tsoukos et al., 2021a). Rating of Perceived Exertion (RPE) was obtained using the Borg RPE Scale (ranging from 0 to 10) and ratings were collected before and immediately after performance of the 4 × 12 s bench press sets in every condition (Figure 1) (Lagally and Amorose, 2007).

### Experimental conditions

During all experimental conditions the participants completed a general warm-up which included 5-min of low intensity cycling (50–60 W), followed by 5-min of upper body dynamic stretching for chest and arms (Tsoukos et al., 2019; Tsoukos et al., 2021a). Subsequently, the participants performed a specific warm-up which included: a) a set of 8 repetitions at 50% of the load that followed (either 60% 1-RM in IPC condition or 90% 1-RM in the PAPE and combined IPC + PAPE conditions) and b) a set of five repetitions at 75% of the load that followed, with 3 min rest intervals between the sets. Warm-up sets with the submaximal loads were performed with a controlled movement velocity in order to limit the development of PAPE and neuromuscular fatigue (Rahimi, 2007; García et al., 2022).

After the general and the specific warm-up, the participants performed four experimental conditions in randomized and counterbalanced order:

1) short-duration IPC (5 min): In this condition, 100%AOP was applied for 5 min on both arms, starting 30 s after the end of the specific warm-up (Figure 1). After 5 min of occlusion, the cuffs were deflated, and the participants rested for 5 min (reperfusion period) before they executed the four bench press sets
2) PAPE protocol: In this condition the participants performed a set of 3 repetitions at 90% of 1-RM, within 30 s after the end of the specific warm-up. Then, the participants rested for 5 min before executing the four bench press sets,
3) combination of PAPE and IPC: In this condition, participants performed a bench press set of 3 repetitions at 90% of 1-RM on a Smith machine, within 30 s after the end of the specific warm-up, followed by 5 min of IPC at 100%AOP. Then, the participants rested for 5 min before executing the four bench press performance sets,
4) control condition (CTRL): During the CTRL condition, the participants performed the general and specific warm-up, and then rested for 10.5 min before executing the four bench press performance sets (Figure 1).

Each of the four bench press performance sets lasted 12 s, and the load was 60% of 1-RM, while a 2 min rest was applied between sets. All bench press sets were executed on a Smith machine with a pre-determined grip width. During each set subjects were instructed to move the barbell as fast as possible during both the concentric and eccentric phases. The time was measured by an electronic countdown timer and when the 12th second was reached the participants were informed to stop the movement by a loud audio signal generated by the timer. A spotter assisted the subject to stop the movement by grasping the barbell on the timer signal. If a repetition was stopped during the eccentric phase or at the start of the concentric phase, it was not considered for data analysis.

### Statistical analysis

All results are presented as mean ± standard deviations (SD). Statistical analyses were conducted using the SPSS v. 23 (IBM-SPSS Inc. Armonk, New York, United States). Differences between the four conditions were examined using two-way repeated measures ANOVA (4 conditions x 4 sets). Statistical significance was set at *p* < 0.05. A Tukey’s post hoc test was performed when a significant main effect or interaction was observed. Partial eta square (η^2^) value was used to evaluate the effect size for the interactions and main effects. Partial eta squared values were classified as large (> 0.137), moderate (0.06–0.137) and small (0.01–0.059). For pairwise comparisons, the effect size (ES) was determined by Hedges’ g (small, < 0.3; medium, 0.3–0.8; and large, > 0.8).

## Results

### Average of mean barbell velocities (AMV) in each set

**FIGURE 2:**
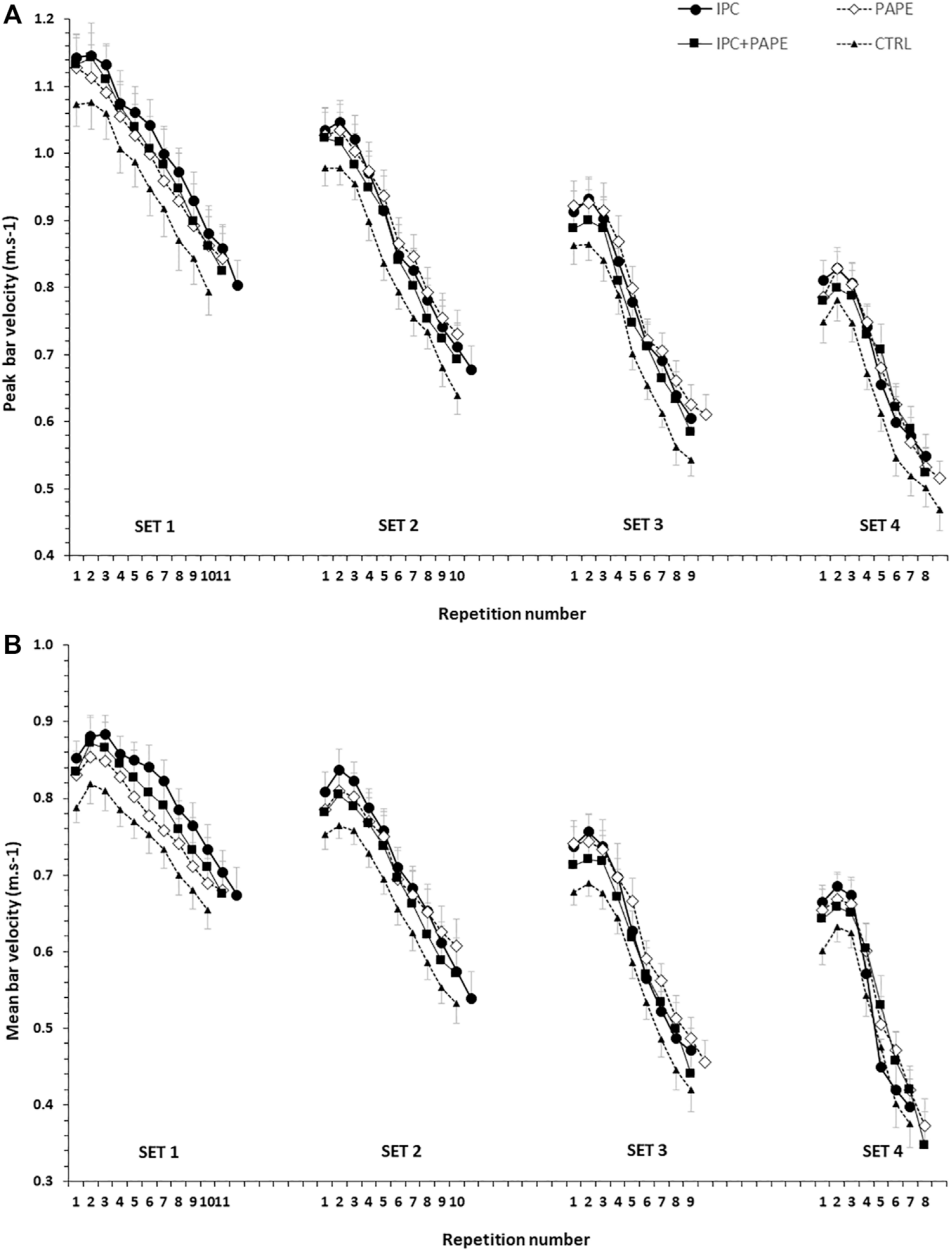
Peak (upper panel) and mean bar velocities (lower panel) per repetition in the four sets (set 1 to set 4) of each experimental condition (CTRL: control, IPC: ischemic pre-conditioning, PAPE: post-activation performance enhancement, PAPE + IPC: combination of PAPE and IPC conditions).

The time-course of changes in peak and mean bar velocity per repetition and set in the four experimental conditions is presented in Figure 2 for visual inspection. Regarding the comparisons of AMV, the 2-way ANOVA revealed a significant interaction (*p* = 0.009, η^2^ = 0.19). Tukey post hoc tests showed that AMV significantly decreased from set 1 to set 4 (set 1 > set 2 > set 3 > set 4; *p* < 0.01) (Table 1) in all four conditions. Post-hoc tests showed that AMV in set 1 was higher in IPC compared with CTRL (+9.0 ± 4.0%) and with PAPE (+4.4 ± 8.9%) (*p* < 0.01; g = 0.77 and *p* < 0.05, g = 0.32, respectively). Also, AMV in set 1 was higher in PAPE + IPC (+5.8 ± 10.0%) compared with CTRL (*p* < 0.01 and g = 0.39). In sets 2 and 3, AMV was higher in IPC (set 2: by 7.0 ± 5.9%, *p* < 0.01 and set 3: by 6.6 ± 5.5%, *p* < 0.05; g = 0.68 and g = 0.64) and PAPE (set 2: by 6.7 ± 10.8%, set 3: by 8.9 ± 12.6%, *p* < 0.01; g = 0.51 and g = 0.74) compared with CTRL. During the fourth set, only PAPE was significantly higher compared with the CTRL (set 4: by 7.6 ± 9.7%, *p* < 0.05, g = 0.64).

**TABLE 1:** The average mean barbell velocities (AMV) in each experimental condition. CTRL: control; IPC: ischemic pre-conditioning; PAPE: post-activation performance enhancement; PAPE + IPC: post-activation performance enhancement and ischemic pre-conditioning.

AMV (m∙s^−1^)				
Condition	SET 1	SET 2	SET 3	SET 4
CTRL	0.74 ± 0.07	0.66 ± 0.06*	0.57 ± 0.06*	0.49 ± 0.06*
IPC	0.80 ± 0.08#‡	0.71 ± 0.08*#	0.61 ± 0.06*†	0.52 ± 0.07*
PAPE	0.77 ± 0.10	0.70 ± 0.09*#	0.62 ± 0.07*#	0.53 ± 0.06*†
PAPE + IPC	0.78 ± 0.12#	0.68 ± 0.12*	0.59 ± 0.10*	0.52 ± 0.09*

*: *p*<0.01 from the previous set; # and †: *p* < 0.01 and *p* < 0.05 from CTRL; ‡: *p* < 0.05 from PAPE.

### Average of peak barbell velocities (APV) in each set

**FIGURE 3:**
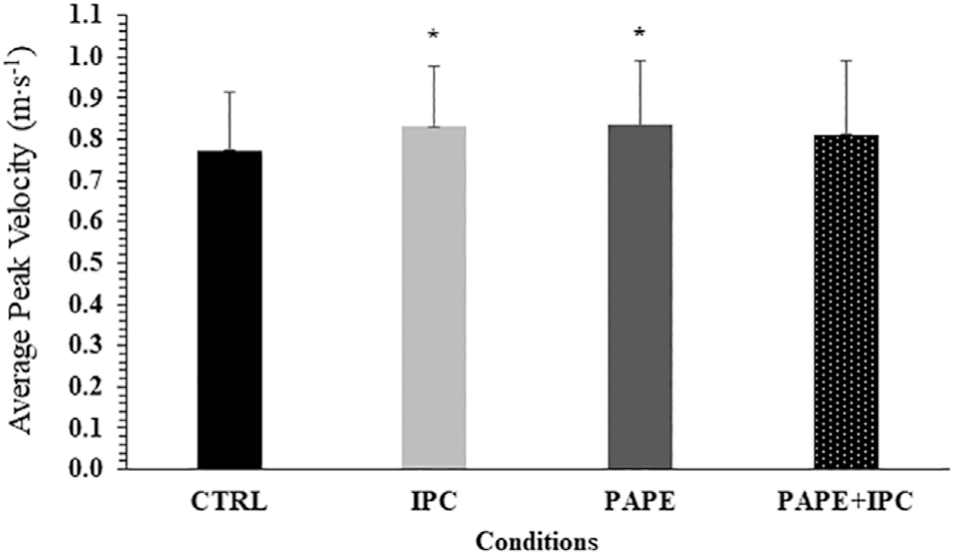
Average peak bar velocities (APV) in each experimental condition (CTRL: control, IPC: ischemic pre-conditioning, PAPE: post-activation performance enhancement, PAPE + IPC: combination of PAPE and IPC conditions). *: *p* < 0.05 from CTRL.

No significant interaction was observed for APV (*p* = 0.98, η^2^ = 0.02). However, the two-way ANOVA showed a significant main effect for condition (*p* = 0.02, η^2^ = 0.25) and set (*p* < 0.001, η^2^ = 0.91). APV significantly decreased from set to set (*p* < 0.001, g = 0.91–2.77). Tukey’s post hoc tests revealed that APV was higher during IPC (+7.8 ± 7.7%, *p* = 0.044, g = 0.40) and PAPE (+8.5 ± 9.6%, *p* = 0.026, g = 0.40) compared with CTRL irrespective of the set (Figure 3).

### Number of repetitions

**FIGURE 4:**
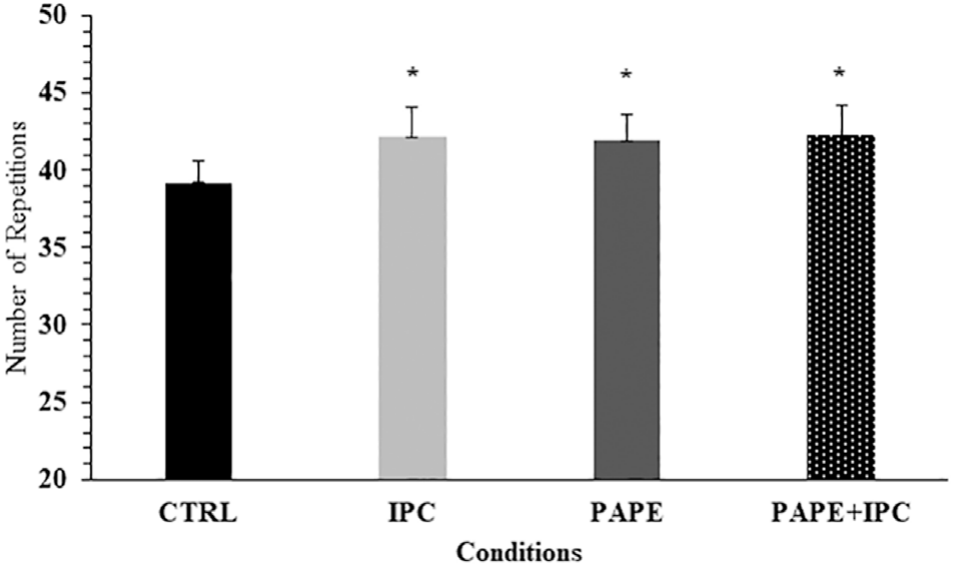
Total number of repetitions (all sets included) in each experimental condition (CTRL: control, IPC: ischemic pre-conditioning, PAPE: post-activation performance enhancement, PAPE + IPC: combination of PAPE and IPC conditions). *: *p* < 0.05 from CTRL.

No significant interaction was observed for the number of repetitions in each set (*p* = 0.10, η^2^ = 0.13). However, the two-way ANOVA showed a significant main effect for condition (*p* = 0.008, η^2^ = 0.30) and set (*p* < 0.001, η^2^ = 0.86). Tukey’s *post hoc* tests revealed that the total number of repetitions was higher during IPC (+7.6 ± 9.5%, *p* = 0.019, g = 0.42), PAPE (+7.4 ± 11.3%, *p* = 0.036, g = 0.41) and PAPE + IPC (+8.0 ± 11.5%, *p* = 0.016, g = 0.43) compared with CTRL (Figure 4). There was also a time effect, showing that number of repetitions significantly decreased from set to set (*p* < 0.01, g = from 0.59 to 2.33).

### Rating of perceived exertion (RPE)

**FIGURE 5:**
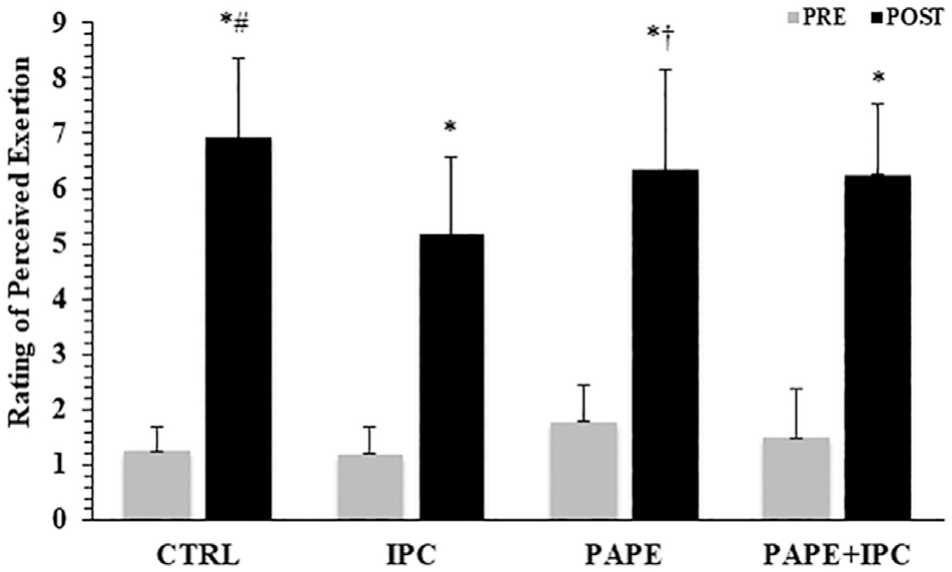
Rating of perceived exertion (RPE) before and after the execution of the four sets in the bench press exercise in each experimental condition (CTRL: control, IPC: ischemic pre-conditioning, PAPE: post-activation performance enhancement, PAPE + IPC: combination of PAPE and IPC conditions). *: *p* < 0.05 from PRE, # and †: *p* < 0.01 and *p* < 0.05 from IPC.

The 2-way ANOVA revealed a significant interaction for RPE (*p* = 0.016, η^2^ = 0.27). Tukey *post hoc* tests showed that RPE after the completion of the four bench press performance sets was lower in IPC compared with CTRL (*p* < 0.001; g = 1.19) and with PAPE conditions (*p* = 0.045; g = 0.69, Figure 5). There was also a trend for PAPE + IPC to be higher than IPC (*p* = 0.076; g = 0.78).

## Discussion

The aim of the present study was to examine the acute effects of short-duration IPC (1 cycle of 5 min at 100% of full arterial occlusion pressure (AOP) and a very low-volume, high-resistance PAPE protocol (1 set of 3 repetitions at 90% of 1-RM in the bench press exercise), as well as their combination (PAPE + IPC) on performance during repeated sets of the bench press exercise. The main finding of the present study was that short-duration IPC, PAPE and their combination resulted in similar overall improvements in bench press performance compared with CTRL condition. However mean barbell velocity was improved in first set only in IPC. Interestingly, the improvement in performance after IPC was accompanied by almost 2 a. u. Lower RPE than CTRL (IPC: 5.2 ± 0.5 vs. CTRL: 6.9 ± 0.5, *p* < 0.001; g = 1.19), indicating a beneficial effect of IPC on performance vs perceived effort relationship. The PAPE protocol was as effective as IPC only in sets 2-4, while AMV in set 1 was higher in IPC. Also, RPE in PAPE was not different from CTRL. The combination of PAPE + IPC resulted in improved AMV only in set 1, while performance in subsequent tests did not differ from CTRL, possibly due to prevalence of fatigue over performance enhancement.

To our knowledge this is the first study examining the effects of short-duration IPC on the volume of training and performance of repeated bench press sets. The beneficial effects of such a short-duration IPC are of great practical interest, as they can be easily applied to training. Only a short period of time (10 min) is required to potentiate performance during repeated bouts of the bench press exercise, compared with the previous studies where a total of 30–40 min at much higher occlusion pressures (e.g., 220 mmHg) were required to induce beneficial effects on resistance exercise performance (Carvalho and Barroso, 2019; Guilherme Da Silva Telles et al., 2020; da Silva Novaes et al., 2021). Thus, it is evident that a single 5-min cycle of IPC is adequate to induce significant increases in mean barbell velocity (from 6.6 ± 5.5% to 9.0 ± 4.0%) and total number of repetitions (7.6 ± 9.5%), compared with the CTRL condition, and so practitioners may easily apply it in exercise training. So far, only a limited number of studies have examined short duration IPC, and this was on single effort explosive muscle performance (Beaven et al., 2012). For example, two short bouts of IPC (2 × 3 min) applied on the thighs, resulted in a (9.0 ± 9.1%) improvement in jump height (Beaven et al., 2012).

Research has shown conflicting results regarding the effects of IPC on subsequent performance. Some authors found no difference between the IPC protocol (220 mmHg of cuff pressure) and a sham condition (20 mmHg of cuff pressure) (Marocolo et al., 2016a; Marocolo et al., 2016b), while others reported acute increases in strength and power performance (Paradis-Deschênes et al., 2016; de Oliveira Cruz et al., 2019; Jarosz et al., 2021; Wilk et al., 2021). For example, Marocolo et al. (Marocolo et al., 2016a) evaluated the effects of IPC (4 × 5-min occlusion at 220 mmHg) and a sham condition on resistance exercise performance in the lower and upper limbs and found that both IPC or sham may enhance performance during resistance exercise with no difference between them. The authors concluded that the same number of repetitions performed by both groups (IPC or sham) were due to the higher psychological motivation and could not be attributed to physiological mechanisms. In contrast, several studies have shown a beneficial effect of IPC, compared with sham and control conditions. Paradis-Deschênes et al. (Paradis-Deschênes et al., 2016) found an increase in peak and average force after IPC (3×5-min IPC/5-min reperfusion cycles at 200 mm Hg) compared with a sham condition, while Wilk and colleagues (Wilk et al., 2021) showed that ischemia (80% of AOP) applied between five sets of bench press exercise at 60% 1-RM, enhanced peak velocity and peak power during the third, fourth and fifth set by 7.5%, 7.3%, 8.6% respectively, compared with the control condition (Wilk et al., 2021). These improvements may be attributed to acute increases in: 1) neuromuscular activation (overall sEMG amplitude), 2) the accumulated oxygen deficit, 3) the amplitude of blood lactate kinetics, 4) the total amount of oxygen consumed during recovery, 5) muscle deoxygenation kinetics, 6) oxygen uptake (VO2) peak (de Oliveira Cruz et al., 2017) 7) muscle oxygenation and 8) phosphocreatine (PCr) resynthesis (Andreas et al., 2011). Regarding the neuromuscular activation, de Oliveira Cruz et al. (de Oliveira Cruz et al., 2016, 2019) observed a parallel increase in performance and electromyographic activity after an intermittent bilateral cuff inflation [4 × (5 min of blood flow restriction + 5-min reperfusion)] (de Oliveira Cruz et al., 2016, 2019). Higher neural activation may be caused by an increase in concentration of metabolic byproducts, such as hypoxia-inducible factor 1a (HIF 1a), opioid peptides, endogenous cannabinoids and other factors which may cause a decrease in the activation of the group III and IV muscle afferent fibers (de Oliveira Cruz et al., 2017). Ischemia may also trigger an increase in ATP production by glycolytic and phosphagen paths (Janier et al., 1994; Mendez-Villanueva et al., 2012). The physiological basis of the increase in movement velocity may be explained by changes in the metabolic substrates and energy metabolism (Kraemer et al., 1987; Robergs et al., 1991). In the present study we observed a significant difference between the CTRL condition and IPC in AMV from the first until the third set. This greater maintenance of AMV during the IPC condition may be due to an increase in blood volume which is observed after the use of IPC (Cunniffe et al., 2017). It has also been shown that higher blood flow caused by higher concentration in adenosine and nitric oxide that leads to opening of K_ATP_ potassium channels which result in greater vessel diameter (Rosenberry and Nelson, 2020). The higher blood flow might cause greater oxygen supply to the muscle and thus a faster PCr resynthesis (Hogan et al., 1999). Faster PCr resynthesis is critical in exercise performance, particularly during repeated efforts of high intensity contractions with incomplete recovery (Bogdanis et al., 1996; Mendez-Villanueva et al., 2012).

Another fact that merits discussion regarding the results of the present study was that ischemic preconditioning was applied distally to the pectoralis major and anterior deltoid muscles, which are among the prime movers of the bench press exercise. The positive effect of such cuff placement has been considered “a paradox” and may be induced by a greater muscle activation of the muscles that are proximal to the pressure cuffs. (Yasuda et al., 2010; Hedt et al., 2022).

The higher total number of repetitions observed in our study is in line with previews studies which found a 12–17% increase following several-fold longer IPC (Paradis-Deschênes et al., 2016; Tanaka et al., 2016). This improvement may be due to increased muscle oxygenation and muscle blood volume (45,56), implying higher blood flow to the exercising muscle (Incognito et al., 2016), which may also lead to faster removal of metabolic byproducts and lower peripheral fatigue (Amann and Calbet, 2008). Furthermore, an increase in muscle blood flow and water content of the muscle has been proposed as one of the main physiological mechanisms of PAPE which has been shown to substantially enhance muscle force and shortening velocity (Blazevich and Babault, 2019). This might be the reason of the greater total volume of training. The higher total number of repetitions in PAPE and PAPE + IPC may be beneficial during systematic training when the aim is to maximize muscle hypertrophy (Schoenfeld et al., 2021), by inducing greater anabolic intracellular signaling (Terzis et al., 2010), higher protein synthesis rate (Burd et al., 2010) and enhanced satellite cell responses (Hanssen et al., 2013).

Alongside with the greater mean and peak barbell velocity, which was observed in IPC, there was also a lower RPE. These results are in line with a previous study during maximal constant-load cycling, which reported lower RPE after 4 × 5 min high pressure IPC (220 mmHg), and a parallel increase in peak VO_2_, faster oxygen kinetics and higher vastus lateralis sEMG activity (de Oliveira Cruz et al., 2015). Furthermore, a recent study examining the origin of fatigue during an intermittent isometric protocol of the knee extensor muscles at 40% of MVC till exhaustion, found that the ergogenic effect of IPC has a neural origin which lowers neuromuscular fatigue (Pethick et al., 2021). Therefore, the improvement in performance and the lower RPE observed in the present study following IPC may be explained by the above-mentioned mechanisms.

The participants of the present study achieved higher mean (from 6.7 ± 10.8% to 8.9 ± 12.6%) and peak (8.5 ± 9.6% overall irrespective of the set) velocities in the bench press exercise after the PAPE protocol compared with the control condition. This finding confirms previous results and demonstrates the beneficial effects of very low volume, high-intensity conditioning exercise on subsequent performance during training and competition in different sports, such as track and field, gymnastics and team sports (Jemni et al., 2006; Takanashi et al., 2020; Gonçalves et al., 2021). Although the effects of PAPE in power and movement velocity are well known (Tsolakis et al., 2011; Tsoukos et al., 2021a), few studies have investigated the influence of this method on the total number of repetitions (Alves et al., 2019; Krzysztofik et al., 2020). Alves et al. (Alves et al., 2019) used a similar conditioning protocol with the present study (3 repetitions at 90% of 1-RM) and found an increase in the number of repetitions performed during three sets of the bench press against 75% 1-RM to failure with 1.5-min rest interval between sets (Alves et al., 2019). In contrast, Krzysztofik and colleagues (Krzysztofik et al., 2020) examined the effects of a PAPE protocol on resistance training volume during the bench press exercise and found slightly different results compared with our study. Specifically, the authors did not find a statistically significant difference between the PAPE protocol and the control condition for barbell velocity and the number of repetitions performed, although a greater total time under tension was found (Krzysztofik et al., 2020). The lack of an increase in barbell velocity and the number of repetitions in that study may be due to the larger volume of the PAPE protocol used, i.e., 3 sets of 3 repetitions at 85% of 1-RM (Krzysztofik et al., 2020), which was 3-fold higher than that used in the present study. Thus, the prevalence of fatigue may outweigh the beneficial effects of PAPE on total number of repetitions, lending further support to the use of very low volume, high-intensity protocols for optimal results.

This study is the first to examine the effect of a combined PAPE and IPC protocol (PAPE + IPC) on performance and volume of training. The combined protocol induced an increase in mean barbell velocity compared with CTRL only in the first of the four sets (Table 1), while peak barbell velocity was unaffected. In contrast when PAPE or IPC were applied, we observed higher barbell velocities compared with CTRL. A possible explanation may be that the combination of PAPE + IPC resulted in greater muscle fatigue, which counteracted the positive effects of each intervention on barbell velocity. However, the effects of PAPE + IPC on total number of repetitions were similar to the other interventions, and therefore cumulative and not peak performance was enhanced by this combination of pre-activation protocols. A limitation of the present study is that we did not use the inverse order of interventions in the combined condition. That is, to use first the IPC and then the PAPE set. This combination could have resulted in improvements in barbell velocity and should be examined in future studies, along with the physiological mechanisms involved.

In conclusion, short duration IPC (5 min), using a relatively moderate cuff pressure (i.e., 100% AOP; 146.7 ± 15.0 mmHg) enhanced performance during repeated sets of the bench press exercise, by inducing increases in mean barbell velocity (AMV: from 6.6 ± 5.5% to 9.0 ± 4.0%) and total number of repetitions (by 7.6 ± 9.5%) of the session. Notably, this improvement in performance after IPC was accompanied by lower perception of effort (i.e., lower RPE than CTRL). In addition, the PAPE protocol less effective than IPC in set 1, but equally effective in sets 2-4, while RPE was higher, and similar to CTRL. The combination of PAPE + IPC resulted in improved mean barbell velocity only in set 1, while performance in subsequent tests did not differ from CTRL, possibly due to prevalence of fatigue over performance enhancement. Compared to CTRL, all interventions resulted in improved total number of repetitions during the repeated sets of bench press exercise, executed as fast as possible against 60% 1-RM. Due to its brief duration and lower discomfort and perceived exertion, short-duration IPC may be used to enhance power output during training and competition requiring fast repeated muscle actions, as well as when athletes aim to maximize total training volume during muscle hypertrophy protocols.

## Data Availability

The raw data supporting the conclusions of this article will be made available by the authors, without undue reservation.
